# Editorial: Crosstalk between lipid metabolism and ferroptosis in cardiovascular diseases

**DOI:** 10.3389/fcvm.2023.1296935

**Published:** 2023-10-05

**Authors:** Yuliang Liu, Yingying Guo, Pengyong Li, Haipeng Guo, Man Xu

**Affiliations:** ^1^ Department of Critical Care Medicine, The Key Laboratory of Cardiovascular Remodeling and Function Research, Chinese Ministry of Education and Chinese Ministry of Health, Qilu Hospital, Cheeloo College of Medicine, Shandong University, Jinan, China; ^2^ Department of Cardiology, Renmin Hospital of Wuhan University, Hubei Key Laboratory of Metabolic and Chronic Diseases, Wuhan, China

**Keywords:** ferroptosis, cell death, lipid metabolism, lipid peroxidation, cardiovascular disease

**Editorial on the Research Topic** Crosstalk between lipid metabolism and ferroptosis in cardiovascular diseases

Cardiovascular diseases (CVDs) pose a significant threat to human health, with their prevalence steadily increasing even among younger populations in developed countries (1). Moreover, CVD is still the primary cause of death in China, the world’s largest developing country (2). Therefore, devoting more attention to the pathogenesis and therapeutic measures of CVDs is crucial for maintaining health and reducing economic burden. The myocardium is not characterized by self-renewal, so the function and survival of cardiomyocytes are of particular concern in cardiovascular health. In recent years, ferroptosis has gradually been recognized as an essential type of death in cardiomyocytes and is usually initiated by lipid peroxidation. The metabolic pathways of ferroptosis mainly involve iron, lipid, and amino acid metabolism. Mitochondrial activity and intracellular redox homeostasis are also significantly associated with ferroptosis. Extensive previous studies have demonstrated a close association between different components of lipid metabolic pathways and CVDs. To identify the crosstalk between ferroptosis and lipid metabolism and explore how it affects cardiovascular health, we present new evidence regarding ferroptosis and lipid metabolism in the cardiovascular system, including clinically relevant drug applications, through this editorial. In addition, we summarize the new findings of the contributing articles on this research topic.

Recent studies indicated that iron deficiency may affect the prognosis of some patients with CVDs, and iron supplementation can benefit patients who suffer from heart failure with reduced ejection fraction or mildly reduced ejection fraction (HFrEF/HFmrEF) (3). However, there is growing evidence that cell death triggered by iron overload is deeply engaged in the emergence of many CVDs and a common pathophysiologic process in heart failure, cardiomyopathy, myocardial ischemia/reperfusion injury (I/RI), drug-associated myocardial damage, diabetic cardiomyopathy, and others (4). Teng and colleagues’ bibliometric analysis of ferroptosis in CVDs recently enriched our research topic. Their findings, presented through data visualization, indicate that research in this area has grown steadily in recent years. Specifically, studies have concentrated on I/RI, heart failure, and atherosclerosis. At the same time, they also provide scholars with the research direction of ferroptosis in the future, such as endothelial damage and gut microbiota-related cardiomyocyte ferroptosis. Potential inducers or inhibitors of ferroptosis are also promising research topics for CVDs.

**Figure 1:**
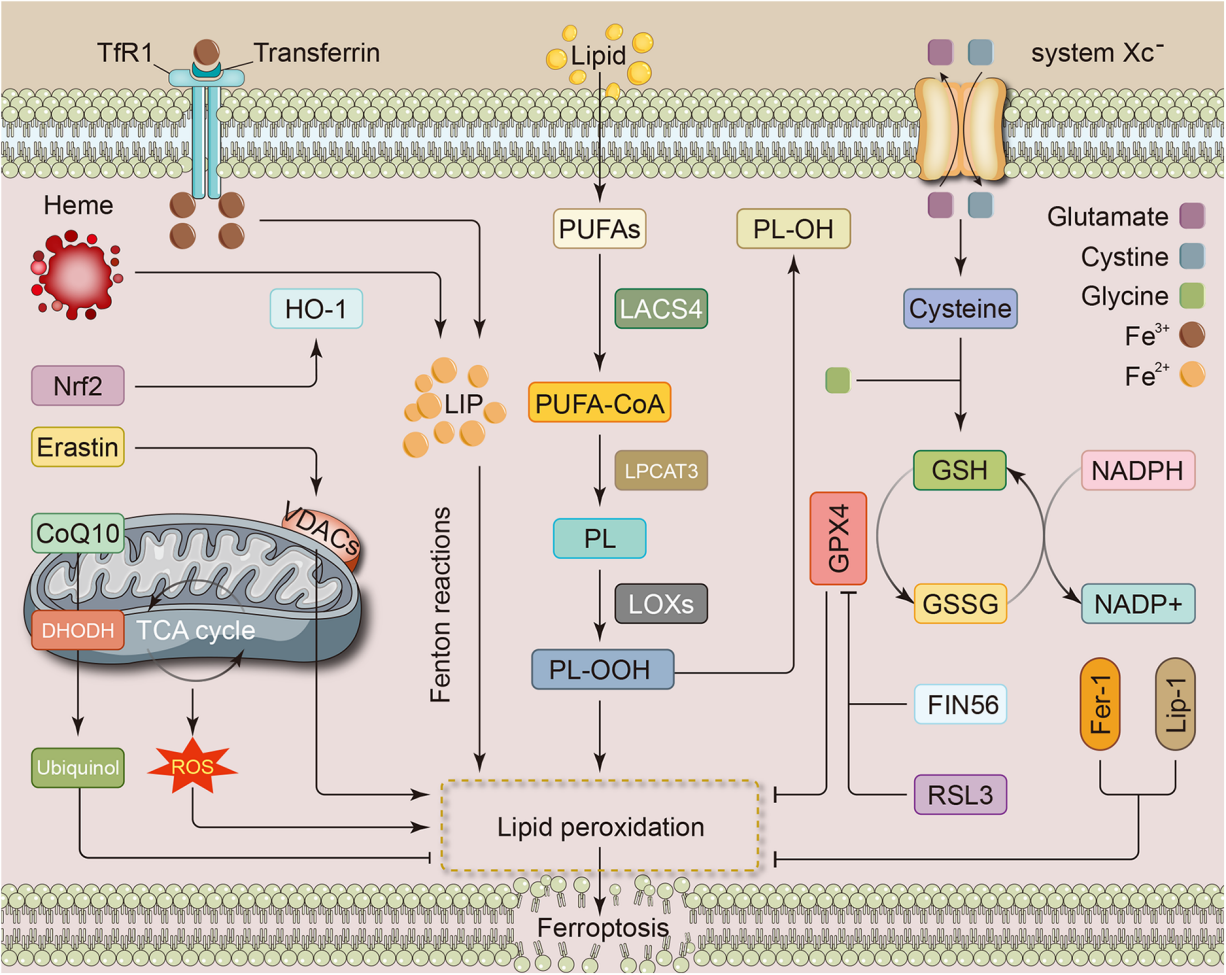
Metabolic pathways associated with ferroptosis in cardiovascular disease. The metabolic pathways of ferroptosis mainly involve iron, lipid, and amino acid metabolism. Iron overload from any cause can be a source of LIP and promote the development of ferroptosis, e.g., nrf2 promotes heme catabolism and thus increases free iron levels. Intracellular PUFAs are converted to toxic forms through a series of enzymatic reactions, which in turn lead to the accumulation of lipid peroxides. The system Xc^—^, consisting of the subunits SLC3A2 and SLC7A11 subunits, is responsible for transporting glutamate and cystine. Cystine undergoes conversion to cysteine and then participates in the synthesis of GSH. GPX4, which plays a role during the transformation of GSH to GSSG, inhibits lipid peroxidation and attenuates ferroptosis. In addition, several metabolic processes within the mitochondria and some substances, such as RSL3 and Fer-1, can also influence the development of ferroptosis through specific mechanisms. CoQ10, coenzyme Q10; DHODH, dihydroorotate dehydrogenase; Fer-1, ferrostatin-1; FIN56, ferroptosis inducing 56; GPX4, glutathione peroxidase 4; GSH, glutathione; GSSG, oxidized glutathione; HO-1, heme oxygenase-1; LACS4, long-chain fatty acid coenzyme A (CoA) ligase 4; LIP, labile iron pool; Lip-1, liproxstatin-1; LOX, lipoxygenase; LPCAT3, lysophosphatidylcholine acyltransferase3; NADPH, nicotinamide adenine dinucleotide phosphate; Nrf2, nuclear factor erythroid 2-related factor; PL, phospholipids; PUFA, polyunsaturated fatty acids; ROS, reactive oxygen species; RSL3, RAS-selective lethal 3; TCA, tricarboxylic acid; TfR1, transferrin receptor 1; VDAC, voltage-dependent anion channel.

Dixon et al. initially identified ferroptosis as a type of regulated cell death (RCD) that can be repressed by ferrostatin-1 and is activated by RAS-selective lethal (RSL) compounds, such as erastin and RSL3. Unlike other forms of RCD, electron microscopic views of mitochondrial outer membrane rupture, cristae reduction, and mitochondrial membrane condensation characterize ferroptosis (5). During amino acid metabolism, cystine is transported into the cell by the glutamate/cystine antiporter system Xc^—^, composed of the subunits SLC3A2 and SLC7A11. The intracellular cystine is then altered to cysteine and is involved in glutathione (GSH) synthesis. As an active peroxidase, glutathione peroxidase 4 (GPX4) rapidly oxidizes GSH to glutathione disulfide (GSSG) and inhibits lipid peroxidation or promotes the degradation of lipid peroxides. GSH depletion or reduced GPX4 activity can lead to impaired lipid peroxide metabolism and thus aggravate ferroptosis (6). Scavenging lipid peroxides reduces oxidative stress in cardiomyocytes and lessens ferroptosis severity (Figure 1).

Lipid metabolism affects phospholipid peroxidation and various other cellular processes associated with it, so lipid metabolism may regulate ferroptosis through various signaling pathways, e.g., by regulating the levels of lipid peroxides and related lipid metabolizing enzymes. Indeed, excess lipid peroxides are considered to be key drivers in ferroptosis (7). The acylation of polyunsaturated fatty acids (PUFA) to toxic forms by the enzyme long-chain fatty acid CoA ligase 4 (LACS4) is a significant process of lipid peroxidation in ferroptosis, makes cells more sensitive to ferroptosis and promotes the initiation of iron-dependent cell death cascade responses (8). In contrast, LACS3 reduces the accumulation of reactive oxygen species (ROS) and lipid peroxides at the plasma membrane by activating exogenous monounsaturated fatty acids (MUFAs) (9). In addition, PUFAs are sequentially converted by lysophosphatidylcholine acyltransferase3 (LPCAT3) and lipoxygenase (LOX) to PUFA-containing membrane phospholipid and lipid ROS, which then mediate the membrane injury (10). As main substances produced by the process of lipid peroxidation, malondialdehyde (MDA) and 4-hydroxynonenal (4-HNE) (11) are also present in cardiac tissues subjected to oxidative stress injury and are down-regulated by the nuclear factor erythroid 2-related factor (Nrf2) activator resveratrol (12). Notably, during doxorubicin (DOX)-induced myocardial injury, increased heme oxygenase-1 (HO-1) induced by Nrf2 promotes heme degradation and free iron release, exacerbating ferroptosis and myocardial injury (13). However, numerous previous investigations have shown that the Nrf2/HO-1 system is essential for ROS scavenging and attenuating oxidative stress injury. Thus, ferroptosis may impair myocardial viability through its unique role, although it is involved in oxidative stress. As represented by acute infarction, ischemic heart disease is also characterized by a high mortality rate, and cell death exhibits key effects in I/RI. Feng and colleagues discovered that liproxstatin-1, a ferroptosis inhibitor, lessened I/RI in mice myocardium by restoring GPX4 concentration, decreasing ROS production and accumulation in mitochondria, preserving mitochondrial function, and reducing myocardial infarction area (14).

As previously described, SLC7A11, one of the subunits of the system Xc^—^, attenuates ferroptosis and cardiovascular injury by promoting GSH synthesis and ROS clearance. Interestingly, Hu et al. showed that in the animal model of pulmonary arterial hypertension, erastin could attenuate pulmonary vascular remodeling by inducing ferroptosis to attenuate the proliferation of pulmonary arterial smooth muscle and subsequently alleviate pulmonary vascular remodeling. The mechanism of action was that erastin administration decreased the levels of SLC7A11 and GPX4 (15). Conversely, Xie et al. concluded that ferrostatin-1, a ferroptosis inhibitor, helped attenuate MCT-induced pulmonary hypertension, restored right ventricular function, and attenuated inflammatory damage to pulmonary artery endothelial cells (16). Recently, Xu and Bu provided new perspectives on Hmox1 and SLC7A11 in CVDs to our research topic. They analyzed two gene expression datasets, GSE185754 and GSE171546, and then identified *Hmox1* (HO-1) and *SLC7A11* as ferroptosis-related targets. Subsequently, the authors used siRNA to knock down these two genes in the model of LPS-induced myocardial injury, demonstrating that downregulation of Hmox1 and SLC7A11 contributes to restoring cardiomyocyte viability and morphology, potentially attenuating septic cardiomyopathy. Disturbed lipid metabolism is a characteristic metabolic abnormality of atherosclerosis. In the high-fat diet-induced atherosclerosis in ApoE-/- mice model, ferrostatin-1 restored the expression of SLC7A11 and GPX4 while also reducing iron content. In addition, inhibition of ferroptosis represented attenuated lipid peroxidation, which was closely associated with improved arterial endothelial function (17).

Researchers have also progressively begun to understand ferroptosis at the genetic level in recent years. Wu et al. discovered several ferroptosis-associated genes, such as *CA9*, *CBS*, *CEBPG*, and *HSPB1*, which are considered to be strongly associated with coronary artery disease (18). In addition, several studies on this topic have identified ferroptosis-related genes, offering new targets for ferroptosis. Li et al. identified genes associated with ferroptosis in calcific aortic valve disease for the first time. In addition, they conducted GSEA and KEGG pathway analyses, followed by validation *in vitro* experiments. Finally, they indicated that *IL-6*, *BID*, *PRKAA2*, *HIF-1*, and *HMOX1* genes are relevant targets for ferroptosis; accordingly, the NAFLD and the HIF-1 pathway act as ferroptosis-related signaling pathways that influence the development of calcific aortic valve disease. Similarly, Huang et al. analyzed gene expression datasets in ischemic cardiomyopathy (ICM) and identified dozens of differentially expressed genes associated with ferroptosis. They also considered *IL-6*, *JUN*, *STAT3*, *MAP3K5*, and *ATM* as the top five hub genes, which were also confirmed to be significantly altered in expression by qRT-PCR in clinical blood samples from ICM. Subsequently, functional enrichment analysis revealed that ferroptosis in ICM may involve oxidative stress, immune-related, and inflammation-related signaling pathways. In summary, further studies are being conducted to identify numerous ferroptosis-related genes, and the mechanisms that affect CVD deserve further exploration.

Based on various preclinical studies, certain substances like ferrostatin-1 have shown effective anti-ferroptosis properties through mechanisms like preventing iron overload, scavenging ROS, activating GPX4, and reducing lipid peroxidation (4). Wang et al. showed that tanshinoneIIA, similar to ferrostatin-1, restored GPX4 and SLC7A11 expression in high-power microwave (HPM)-damaged cardiomyocytes while decreasing the levels of Hmox1 and ACSL4, which ultimately attenuated the severity of HPM-induced ferroptosis. Future studies should focus more on collecting evidence for the role of ferroptosis in human specimens or *in vivo*, sorting out the complex relationship between ferroptosis and lipid metabolism, ultimately providing a basis for the discovery of new markers and the development of novel ferroptosis inducers and/or inhibitors. In addition, the risk factors of ferroptosis in cardiomyocytes, the predictive indicators of ferroptosis, the predictive value of ferroptosis and its degree in different CVDs, such as what degree of ferroptosis can predict the possible recurrence of inflammation and oxidative stress, and the timing or degree of intervention for ferroptosis targets, are all topics that may need to be explored in the future.

Overall, this research topic addresses the role of ferroptosis in CVDs and highlights the diverse mechanisms by which it affects disease progression. Based on the current evidence, we firmly believe that ferroptosis and lipid metabolism have the potential to be effective therapeutic targets. However, more in-depth research is required to clarify the crosstalk between ferroptosis and lipid metabolism, ultimately determining the reliability of its transformation into clinical application. We hope this research topic will not only provide readers with new insights into ferroptosis and cardiovascular health but also inspire new advances in this research field and even other research topics.
